# Precipitation and Transformation of Vaterite Calcium Carbonate in the Presence of Some Organic Solvents

**DOI:** 10.3390/ma13122742

**Published:** 2020-06-17

**Authors:** Donata Konopacka-Łyskawa, Natalia Czaplicka, Marcin Łapiński, Barbara Kościelska, Rafał Bray

**Affiliations:** 1 Department of Process Engineering and Chemical Technology, Faculty of Chemistry, Gdansk University of Technology, Narutowicza 11/12, 80-233 Gdansk, Poland; natalia.czaplicka@pg.edu.pl; 2 Department of Solid State Physics, Faculty of Applied Physics and Mathematics, Gdansk University of Technology, Narutowicza 11/12, 80-233 Gdansk, Poland; marcin.lapinski@pg.edu.pl (M.L.); barbara.koscielska@pg.edu.pl (B.K.); 3 Department of Water and Wastewater Technology, Faculty of Civil and Environmental Engineering, Gdansk University of Technology, Narutowicza 11/12, 80-233 Gdansk, Poland; rafal.bray@pg.edu.pl

**Keywords:** calcium carbonate, carbonation, carbon dioxide sequestration, solvents

## Abstract

In this paper, the production of CaCO_3_ particles via the carbonation route in the reaction of CaCl_2_ and CO_2_, using NH_3_ as a promoter of CO_2_ absorption, was studied. The solvents used as the reaction media for CaCO_3_ precipitation were aqueous solutions of methanol, isopropanol and dimethyl sulfoxide (DMSO), in a concentration range of 0–20% (*v*/*v*). It was found that the presence of an organic additive influenced the precipitation rate, the content of vaterite in the obtained product, the morphology and the size of the precipitated CaCO_3_ particles, as well as the rate of its transformation into calcite. The presence of all added organic solvents reduced the vaterite concentration in the produced CaCO_3_ both at the end of the reaction and after incubation in the reaction medium for 1 h. However, the transformation of vaterite particles into calcite in the tested solutions was slower when the 4 h and 24 h procedures were compared. The interactions of solvents with calcite and vaterite were compared using HPLC tests. DMSO molecules interacted with vaterite particles the most strongly, while the interaction of isopropanol with this polymorph was the weakest. The opposite effect was observed for interactions with calcite particles, and the affinity decreased in the series: isopropanol, methanol, DMSO.

## 1. Introduction

Calcium carbonate can form three anhydrous polymorphs, i.e., calcite, aragonite and vaterite [1]. Of these crystalline phases, calcite and aragonite occur extensively in nature, while vaterite is a metastable form. Vaterite is considered as a precursor to more stable polymorphs because it can be easily transformed by dissolution and re-crystallization processes [2,3]. The crystallization pathways are controlled by thermodynamic or kinetic steps, depending on the activation energy associated with the crystals’ nucleation and growth. When the reaction profile shifts from the thermodynamic to the kinetic path, the formation of vaterite is promoted [4,5]. Despite relatively low stability, vaterite finds many applications, including biomedicine [6,7], cosmetic preparations [8] and paper production [9], as well as environmental protection [10].

The main method of the industrial-scale production of CaCO_3_ is carbonation, which involves the introduction of gaseous carbon dioxide into a calcium hydroxide suspension. The high pH of the Ca(OH)_2_ suspension facilitates CO_2_ absorption [11]. In this process, calcite is formed at moderate temperatures while aragonite is precipitated at elevated temperatures. Calcium carbonate may also be produced in a liquid–liquid system. This process consists of the mixing of aqueous solutions of soluble calcium salts and carbonates [12]. It is often used in laboratory tests due to the ease of controlling the process variables [13]. Setting appropriate process parameters, such as reagent concentration, temperature or pH, allows researchers to produce vaterite, calcite and aragonite particles [2,14]. Recently, as an alternative method, the carbonation of aqueous solutions of calcium salts in the presence of an absorption promoter, e.g., ammonia or amine, has been proposed [11,15,16]. In contrast to the carbonation of a Ca(OH)_2_ suspension, this method can be used to obtain calcium carbonate in the form of vaterite. This is possible when precipitation is carried out at moderate temperatures with a proper selection of reagent concentrations and a type of absorption promoter [15,17].

One of the factors which has a significant impact on the precipitation of calcium carbonate is the presence of additional substances [18,19,20]. Most often, these additives are not embedded in the formed crystals, but they affect the course of the reaction and the characteristics of the resulting product [1,21,22]. This paper focuses on the influence of the addition of organic solvent into the reactive mixture on the precipitation and stabilization of vaterite.

Research on the influence of solvents on calcium carbonate precipitation are mainly carried out in liquid–liquid systems. Published studies showed that the addition of organic solvents changes the viscosity, density, dielectric constant and surface tension of a reactive mixture [23,24]. It also affects the solubility of the ionic substance and the generation and structure of the absorption layer at the surface of the CaCO_3_ crystals [23]. Important factors influencing the precipitation are the type and number of polar functional groups in the solvent molecules and the presence of hydrophilic and hydrophobic regions [25]. The influence of organic solvents on the mechanisms and the rate of the precipitation steps can consequently lead to changes in the crystal size distribution, polymorph composition and crystal morphology [20,23,26,27,28].

The course of precipitation and the product characteristics depend on many process variables, such as supersaturation, temperature, pH, calcium source, precipitation method, reaction time, mixing intensity and the type of co-solvent [1,22,28,29]. Thus, it is difficult to compare the available research results on the effect of the solvent composition on the crystal formation process.

The problem of calcium carbonate precipitation is much more complex in the gas–liquid system, when CO_2_ absorption is a significant factor influencing the formation of the CaCO_3_ crystal phase. The generation of supersaturation in the solution, and, in consequence, the induction of nucleation and the growth of calcium carbonate particles, depends on the transfer of carbon dioxide from the gas into the liquid phase. The CO_2_ absorption is the slowest step among the series of stages that occur during CaCO_3_ precipitation in the gas–liquid system. Therefore, its control is a key element of producing a product with the required characteristics. The presence of a co-solvent may promote or inhibit the absorption process [26]. Changes in the composition of the aqueous phase result in changes in the solubility of the carbon dioxide, which affects the driving force of absorption [30]. The rate of CO_2_ absorption into the aqueous solution influences the rate of the chemical reaction between carbonate and calcium ions. This rate of absorption depends on parameters such as mass transfer coefficient, equilibrium and actual solubility of CO_2_ and gas-liquid area. When the absorption of carbon dioxide is accelerated, the rate of precipitation increases [30], and the formed primary crystals are smaller [31]. The mass transfer coefficient is inversely proportional to the surface tension and increases as the viscosity of the liquid decreases [23].

In our research, we focused on the possibility of controlling calcium carbonate precipitation by selecting the solvent composition, the interaction of the organic solvent selected as an additive with calcite and vaterite and the impact of the solvent on the vaterite stabilization. The research was carried out using the gas-liquid method with the absorption promoter. The liquid phase involved aqueous solutions of dimethyl sulfoxide (DMSO), methanol or isopropanol. Alcohols are protic solvents that have a so-called acidic proton in their structure that can be broken off by the base molecule. Due to the presence of a highly electronegative oxygen atom and the associated hydrogen atom, alcohols form hydrogen bonds, undergoing association into larger structures [32]. In contrast, DMSO is a strongly solvating aprotic solvent that does not contain acidic hydrogen atoms which can be detached, causing DMSO to have no hydrogen bond donors [24,32]. However, all the selected solvents are polar and have hydrogen bond acceptors. Each alcohol molecule can form three hydrogen bonds, two H-acceptors and one H-donor, while two H-acceptors are present in a single DMSO molecule. Additionally, all the used solvents are completely miscible with water [24]. The selected physicochemical parameters of the water and applied organic solvents are summarized in Table 1.

The aim of this research was to investigate the influence of the addition of selected polar organic solvents on the course of CaCO_3_ precipitation carried out via the carbonation route, as well as to investigate the effect of solvent composition on the polymorphic composition, particle size distribution, morphology and stability.

## 2. Materials and Methods 

### 2.1. Reagents

Anhydrous calcium chloride (≥97.0%, POCH, Gliwice, Poland), 25% ammonia solution (≥96.0%, POCH, Gliwice, Poland), dimethyl sulfoxide (≥99.0%, POCH, Gliwice, Poland), isopropanol (≥99.0%, POCH, Gliwice, Poland) and methanol (≥99.0%, POCH, Gliwice, Poland) were used in this study. The water used to prepare all the solutions was obtained by reverse osmosis filtration.

### 2.2. Particle Preparation

The precipitation process was carried out in a glass tank reactor with a total volume of 1.2 dm^3^, equipped with a magnetic stirrer. The experiments were performed at room temperature and atmospheric pressure. The stirring rate was equal to 800 rpm. The reaction mixture in a volume of 0.8 dm^3^ contained calcium chloride at a concentration of 0.2 mol·dm^−3^, ammonia in a molar ratio of Ca^2+^:NH_3_ equal to 1:1.5 and solvent (DMSO, isopropanol or methanol) at a concentration of 5%, 10%, 15% and 20% (*v*/*v*). Carbon dioxide as a pure gas was supplied into the liquid phase through a sintered diffuser and the gas flow rate was 20 dm^3^·h^−1^ in all experiments. The pH of the mixture was measured every 30 s during the precipitation. An ERH-13-6 composite electrode (HYDROMET, Gliwice, Poland) connected to the pH meter was applied. When the pH was equal to 7, the process was terminated. The Ca^2+^ concentrations were determined every 2 min by the complexometric titration of the collected samples (2·10^−3^ dm^3^) with EDTA (ethylenediaminetetraacetic acid). The obtained suspension was divided into four parts. The first portion was filtered immediately after the completion of the reaction. The CaCO_3_ particles were separated from the remaining portions after 1, 4 and 24 h, respectively. The filtered particles were washed with water and methanol and then dried at 90 °C for 24 h.

### 2.3. Characterization of Particles

To characterize the polymorphic composition of the precipitated calcium carbonate, conventional X-ray diffraction analysis (XRD) with Cu-Kα radiation, using the MiniFlex 600 diffractometer (Rigaku, Tokyo, Japan), was applied. The XRD spectra were collected at room temperature at a scan rate of 0.01° and a 2*θ* angle range of 20 to 80°. Fourier transform infrared spectroscopy (FT-IR) was also applied using the Nicolet 8700 Spectrometer (Thermo Scientific, Waltham, MA, USA), in order to identify the types of chemical bonds. The method of suppressed total reflection (ATR) was used and the spectra were registered from 4500 to 524 cm^−1^ at a 2 cm^−1^ resolution, using air as the background. A FEI Quanta FEG 250 scanning electron microscope (SEM) (FEI, Eindhoven, The Netherlands), equipped with an Everhart–Thornley (ET) secondary electron detector (FEI, Hillsboro, OR, USA), was used to characterize the shape of the precipitated particles. The sizes of the calcium carbonate particles were determined by a laser diffraction method, using the analyzer Mastersizer 2000 (Malvern Instruments Ltd., Malvern, Great Britain) equipped with a standard dispersion unit Hydro 2000MU, with an ultrasonic probe supporting the breaking of agglomerates. The range of measuring the particles’ diameters was between 0.02 and 2000 μm.

### 2.4. Absorption Test

To compare the interaction between the organic solvents and calcium carbonate, normal phase high-performance liquid chromatography (NP-HPLC) was applied. Absorption tests were performed as described earlier [26]. In the current experiment, CaCO_3_ particles in the form of vaterite and rhombohedral calcite as a sorbent were used. A volume of 10 μL of a solution containing 20 mg·mL^−1^ of DMSO, methanol or isopropanol in THF was injected into the tested columns. The capacity factor was determined on the basis of the obtained retention time (*t_R_*) of the individual solvents and the dead time (*t_M_*), using Equation (1).

(1) $$k^{\prime }=\frac{t_{R}-t_{M}}{t_{M}}$$

## 3. Results and Discussion

### 3.1. The Course of the Precipitation

**Figure 1:**
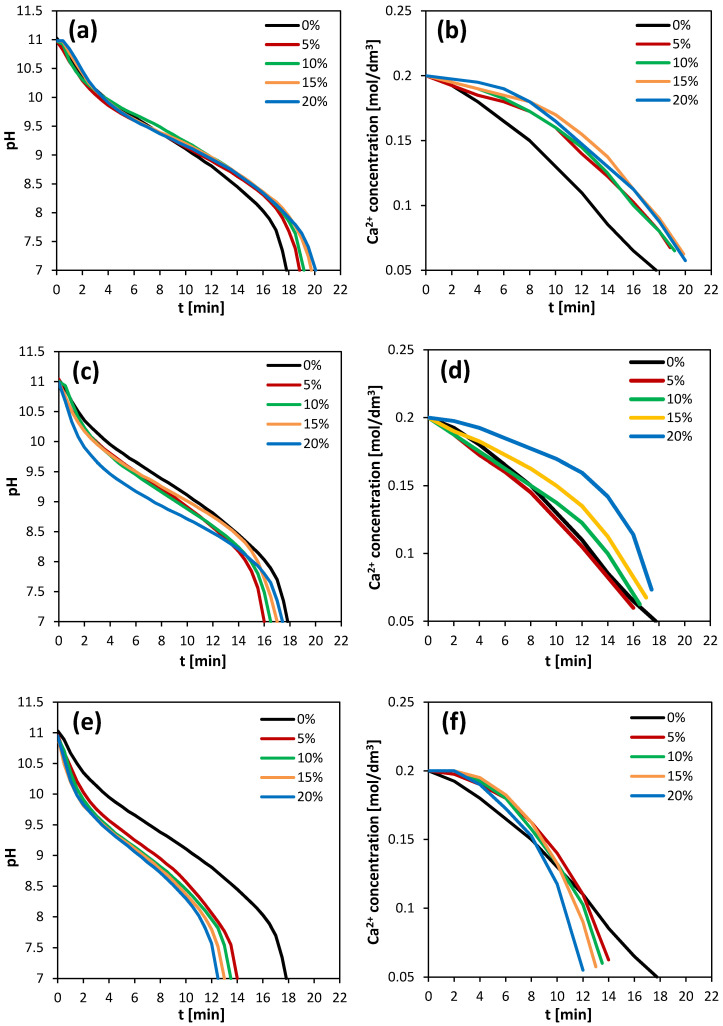
pH and Ca2+ concentration curves versus time for DMSO (**a**,**b**), methanol (**c**,**d**) and isopropanol (**e**,**f**) depending on the solvent concentration.

The course of the precipitation process was monitored by measuring the decrease in pH and the rate of calcium ion consumption. Figure 1 shows the pH curves versus time for all the investigated solvents, depending on their concentration. It can be observed that there was a relationship between the concentration of the selected solvents and the reaction time. An increase in the concentration of DMSO and methanol resulted in an increase in the duration of precipitation, while the opposite trend could be observed when isopropanol was added into the reactive mixture. However, the reactions conducted in the presence of DMSO at each analyzed concentration were characterized by a longer duration compared to the control process without any additives. In turn, the addition of alcohols into the reaction mixture resulted in shorter reaction times compared to the control. Ca^2+^ concentration curves versus time were also obtained. The presence of DMSO caused a slower decrease in the concentration of calcium ions compared to the control sample, although no relationship between the curve [Ca^2+^] = f(t_r_) and the concentration of DMSO was observed, since all the curves had a similar course. For the methanol solutions, an increase in alcohol concentration resulted in a slower decrease in calcium ion concentration. In the case of isopropanol solutions being used as solvents, it was observed that as the alcohol concentration increased, the consumption of Ca^2+^ was faster. Higher final calcium ion concentrations were obtained for all the tested solvents when compared to the control sample. The initial solution’s pH, reaction times and the percentage sequestration of calcium ions in the reaction mixture depending on the concentration of added solvents are summarized in Table 2.

Analyzing the changes in pH during carbonation, it can be observed that the initial intensive pH drop occurred for about 4 min in all the experiments and this can be attributed to the formation of bicarbonate ions in the solution. However, there was no significant decrease in calcium ion concentration in the corresponding period of time. Thus, for this induction time, the required supersaturation in the solution had not yet been formed and the start of the calcium carbonate precipitation was not yet allowed. The concentration of calcium ions began to decrease at the fastest rate in the mixtures containing methanol at 5%, 10% and 15% (*v*/*v*), while the slowest decrease was observed in the case of DMSO. Therefore, by comparing the obtained results to the control process, it can be concluded that the presence of methanol in a concentration below 15% (*v*/*v*) and all tested isopropanol solutions resulted in the accelerated generation of supersaturation in the solution, while the addition of DMSO slowed down this process.

The observed course of calcium carbonate precipitation via the carbonation route depended on the CO_2_ absorption in the reaction mixture. The addition of methanol could facilitate the transport of CO_2_ from the gas phase to the liquid phase. It was found that methanol increased the CO_2_ partitioning into the aqueous phase [33]. Additionally, it was reported that the addition of DMSO in a concentration range from 2.5 up to 3.3 M affected CO_2_ absorption and increased the rate of CO_2_ absorption in the presence of methyldiethanolamine [34]. However, our results obtained in the presence of ammonia did not show a rapid decrease in pH during CO_2_ absorption in the DMSO solutions. The reason for this may be a smaller degree of the dissociation of carbonic acid in the DMSO solutions [34], which led to a slowdown in the consumption of ammonia ions produced in the reaction mixture. Moreover, methanol used as an additive in the reaction mixture caused a decrease in the rate of calcium carbonate precipitation. What is more, calcium carbonate precipitation occurred more slowly in the DMSO solutions compared to the control reaction, while a significant increase in the precipitation rate was observed in the isopropanol solutions. It was found that both low molecular alcohols [35,36] and DMSO [37] can interact with water molecules and can form structures inside the solutions, e.g., ethanol and water molecules exist as clusters because they are not homogeneously dispersed in ethanol–water mixtures [36], the clathrate-like water is formed around DMSO molecule [37] or water molecules are immobilized near the methyl groups of organic molecules [38]. In such binary solvents, the ions may be selectively solvated by water or alcohol [39,40]. These phenomena may explain the lower solubility of ionic substances in solutions and the increase in the supersaturation tendency [29]. In addition, the viscosity of the DMSO solutions was higher than the viscosity of the alcohol solutions, which resulted in the slowing down of the diffusion of molecules in the DMSO solutions, and thus the observed rate of CaCO_3_ precipitation in these solutions was reduced.

### 3.2. Characterization of the Particles

**Figure 2:**
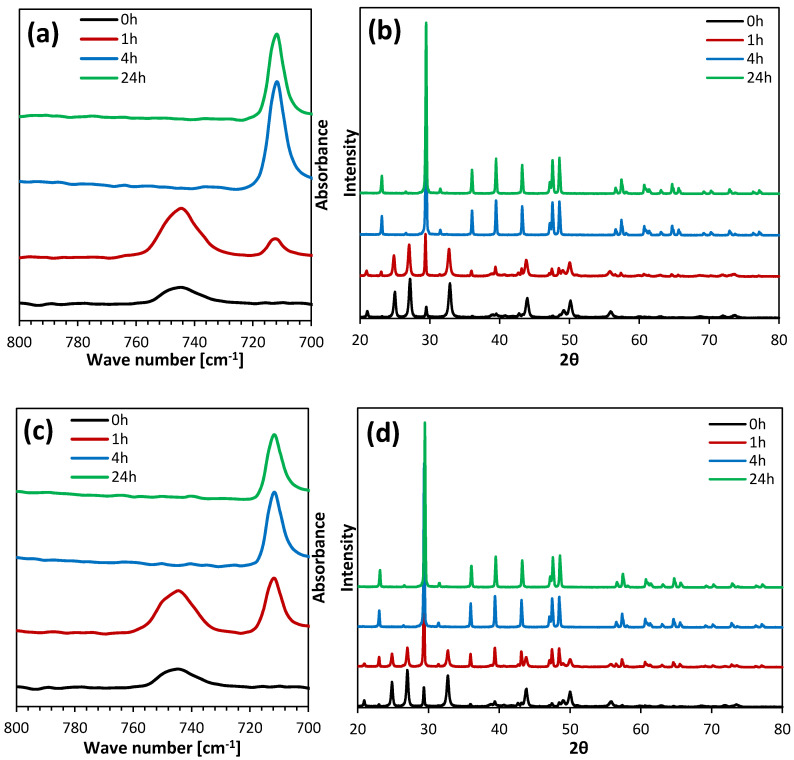
FTIR spectra and XRD patterns of CaCO_3_ precipitated from pure water (**a**,**b**) and from a mixture containing 15% (*v*/*v*) of methanol (**c**,**d**).

The obtained calcium carbonate was analyzed using the ATR-FTIR and XRD techniques to characterize its polymorphic composition. Figure 2 presents the FTIR spectra and diffractograms of the particles precipitated in a control solution without organic solvents in an aqueous solution containing 15% (*v*/*v*) of methanol. The other results are presented in the Supplementary Materials (Figures S1–S4).

**Figure 3:**
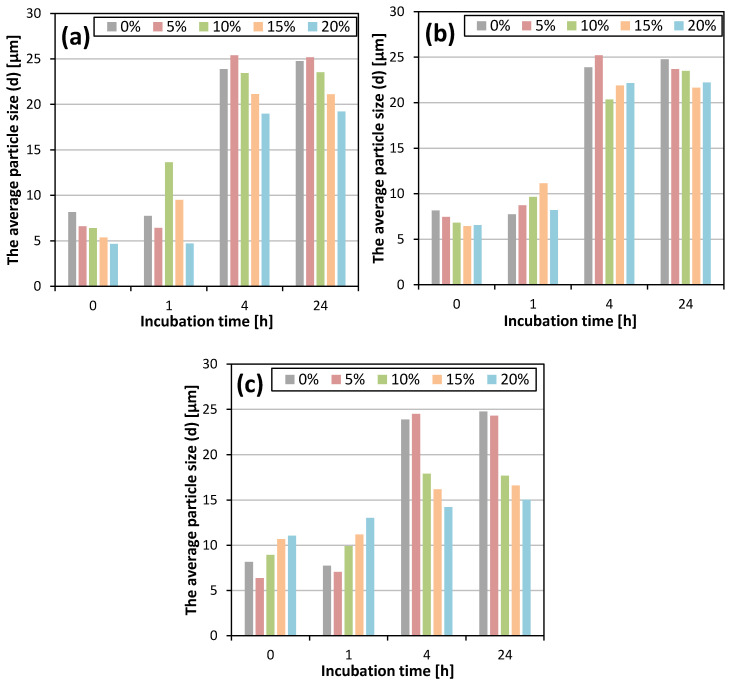
The average particle size (d) depending on the incubation time and solvent concentration for DMSO (**a**), methanol (**b**) and isopropanol (**c**).

In the fingerprint range, characteristic peaks for vaterite and calcite are visible at around 745 and 713 cm^−1^ wave numbers, respectively. However, characteristic vibrations for DMSO should occur at around 1040 cm^−1^, and for alcohols they should occur at around 3200–3500 cm^−1^. No peaks were observed in the recorded ATR-FTIR spectra in these ranges (Figure S4). Thus, the produced dried calcium carbonate did not contain residues of organic solvents. Based on the XRD data, the percentage content of vaterite (*X_V_*) was calculated using Equation (2) [41],

(2) $$X_{V}=\frac{7.691\;\left(I_{V}^{110}\right)}{I_{C}^{104}+7.691\;\left(I_{V}^{110}\right)\;}$$

where I_C_^104^ is the intensity of the calcite peak of the 104 plane and I_V_^110^ is the intensity of the vaterite peak of the 110 plane. It was found that the longer the incubation time, the lower the vaterite content, regardless of the type and concentration of the solvent. This was due to the dissolution of vaterite in the reaction mixture and its recrystallization into more thermodynamically stable calcite [2,3]. The samples that were filtered immediately after the completion of the reaction consisted mainly of vaterite. In this study, the use of all the tested solvents resulted in a lower vaterite content in the samples that were not incubated and incubated for 1 h, compared to the control sample. However, when calcium carbonate was not filtered out and remained in aqueous solutions containing organic solvents, a slowing down of the vaterite recrystallization process was observed, and the content of this polymorph in the obtained products after 4 and 24 h was higher compared to the calcium carbonate samples that were incubated in aqueous solutions without an organic additive. Along with the increase in DMSO concentration, the content of vaterite in the samples that were incubated for 4 and 24 h increased. Samples obtained in the presence of 15% and 20% of methanol showed the largest decrease in the content of vaterite after 1 h incubation. An increase in isopropanol concentration resulted in the production of CaCO_3_ with a lower vaterite content after 0 and 1 h of incubation, while a higher vaterite content was found after 4 h of incubation. X_V_ after 24 h was similar for all the isopropanol concentrations used. Table 3 summarizes the percentage of vaterite content depending on the incubation time and the concentration of the selected solvents. The effect of these parameters on the average particle size is shown in Figure 3 and the particle size distributions (PSDs) are included in the Supplementary Materials (Figure S5).

All the particle size distributions had a similar bimodal course. The first maximum was observed between 0.5 and 0.8 mm, while the position of the second maximum shifted towards higher diameters as the incubation time increased. The relationship between the concentration of the organic solvent and the mean particle size is shown in the Supplementary Materials (Figure S6). For the control sample and all the concentrations of the tested solvents, no significant difference in the average particle size was observed for the samples that were incubated for 4 and 24 h. The influence of additive concentration on average particle size was evident within the first 4 h of incubation (Figure 3). By comparing the sizes of the particles that were separated from the reaction mixture after 4 and 24 h of incubation, the largest increase in particle size occurred when particles were left in a solution without organic additives. In contrast, the smallest increase in crystal size was observed for particles that were incubated in 15% and 20% isopropanol solutions.

**Figure 4:**
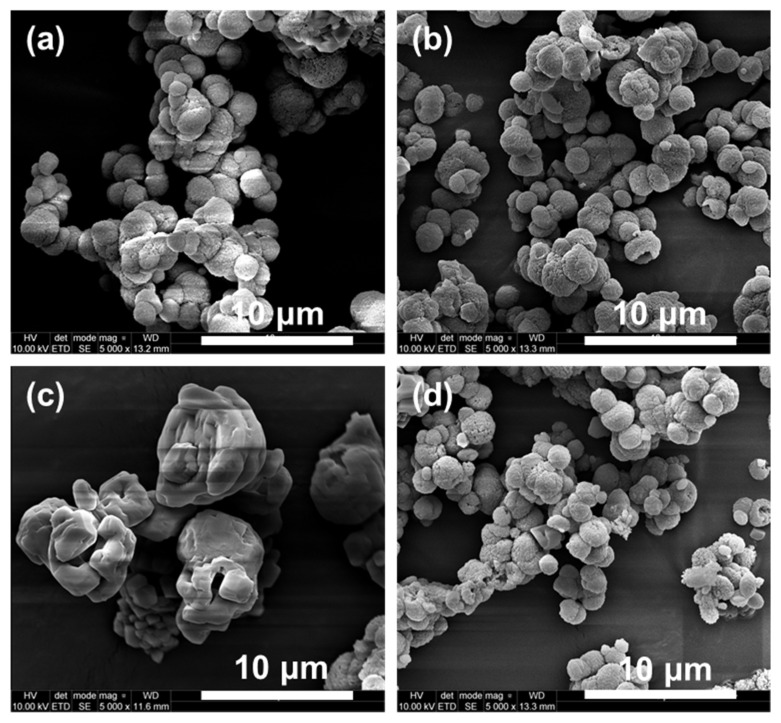
SEM photographs of non-incubated CaCO_3_ particles precipitated without additives (**a**) and in the presence of 15% of DMSO (**b**), isopropanol (**c**) and methanol (**d**).

The obtained calcium carbonate particles were aggregated, irrespectively of the type and concentration of the used solvent. In the case of precipitation from pure water (control), a change in the predominant polymorphic form from spherical vaterite to rhombohedral calcite could be observed with the increasing incubation time, as shown in SEM photographs of the particles (Figure S7 in Supplementary Materials). The presence of DMSO, isopropanol and methanol resulted in the formation of vaterite in the form of spherical particles; however, in the samples that were precipitated with methanol, numerous lens-like particles of this polymorph are also visible (Figure 4d). Calcite was mainly found in the form of strongly aggregated rhombohedral particles with sharp edges. When isopropanol was added into the reactive mixture, aggregated calcite particles with smooth surfaces were observed; however, these did not have pronounced sharp edges (Figure 4c). This phenomenon occurred for 10% and 15% (*v*/*v*) concentrations of isopropanol, while for 5% and 20% concentrations, no such particles were present in the SEM images. Other SEM photographs are attached in the Supplementary Materials (Figures S8–S10).

The precipitation of calcium carbonate in a gas-liquid system occurs in several stages. In the first stage, supersaturation in the solution is achieved as a result of CO_2_ absorption, followed by the subsequent formation of bicarbonate and carbonate ions, reacting with calcium ions present in the system. The next steps involve the formation of a calcium carbonate crystal phase, i.e., nucleation and crystal growth. Depending on the conditions of the process, the resulting crystals can then undergo further transformations as a result of secondary processes, e.g., agglomeration or recrystallization. Under a moderate temperature and a solution pH in the alkaline range, the formation of calcium carbonate particles in the form of vaterite is most often observed [42,43]. Then, as a result of the coupled processes, this form dissolves and recrystallizes into thermodynamically stable calcite [44,45]. The effect of organic additives on the precipitation of calcium carbonate depends on the method of its preparation. When the carbonation of a calcium hydroxide suspension in the presence of ethanol was applied to synthesize CaCO_3_ particles, only calcite was formed in ethanol solutions at a concentration of 0–40% [46]. An increase in organic solvent concentration, up to 100% of ethanol, resulted in the formation of mixed particles containing calcite, aragonite and vaterite polymorphs. However, the addition of ethanol or isopropanol into the reaction mixture used for CaCO_3_ precipitation by a solution method caused the acceleration of the crystal growth rate and the stabilization of the vaterite form by preventing its transformation into calcite [47]. Another experiment using a solution method showed that a mixture of vaterite and calcite was obtained in the presence of 10% ethanol, isopropanol or n-propanol, while aragonite was the dominant form when the alcohol concentration was 50% [22]. Moreover, the polymorphic composition also depended on the intensity of mixing and the residence time in the reaction mixture after the completion of reaction. However, when methanol was a component of the reaction mixture used to obtain calcium carbonate by the diffusion method [48], the precipitated calcium carbonate was mainly calcite for methanol concentrations in the range of 1.75% to 10% and of 30 to 50%, while an increase in the vaterite concentration was observed for moderate methanol concentrations (14–26%). The results obtained in our experiments showed that the addition of an organic solvent to the reaction mixture reduced the proportion of vaterite in the freshly precipitated calcium carbonate particles, even when precipitation occurred faster, i.e., in the presence of isopropanol. Earlier studies on CaCO_3_ precipitation by carbonation in the presence of ammonia indicated that acceleration of the CaCO_3_ precipitation process promotes the formation of vaterite [15,49]. Moreover, vaterite stabilization is observed in systems containing carbamate ions, which can be formed in solutions containing ammonia or primary amines and bicarbonate ions [26,50]. The decrease in the carbamate ion concentrations in the aqueous solutions of organic solvents is affected by reducing the degree of electrolyte dissociation. Therefore, the stabilization of vaterite particles is less effective in these systems.

All organic solvents added to the reaction mixture affected the reduction of carbonate particles that was measured immediately after the completion of the reaction. It is known that a high nucleation rate results in a large population of small particles. In the case of the addition of organic solvents in our study, both a decrease in the degree of dissociation of the substrates was observed and a decrease in the solubility of inorganic substances took place. According to the classical crystallization theory, the rate of nucleation depends mainly on supersaturation, crystal surface energy and temperature. The presence of the tested organic additives increased supersaturation by reducing the solubility of calcium carbonate and reduced the surface energy of the formed crystals.

### 3.3. Interactions of Solvent Molecules with Vaterite and Calcite Particles

The NP-HPLC chromatograms obtained for the investigated solvents in the columns loaded with vaterite and calcite particles are presented in the Supplementary Materials (Figure S11), and the values of calculated capacity factors for tested conditions are collected in Table 4. Since the capacity factor is independent of the column length and flow rate, it is a useful parameter for comparing the results obtained for different systems. The capacity factor is directly related to the strength of the interaction between a solute and the stationary and mobile phases. Among the tested solvents, the retention time in a column filled with vaterite was the longest for DMSO and the shortest for isopropanol. However, in the case of calcite, a reverse relationship was observed. On the basis of these results, it can be concluded that DMSO and methanol molecules interact more strongly with vaterite, while isopropanol molecules have a higher affinity with calcite. The interaction of organic solvent molecules with calcium carbonate particles could be observed when thermodynamically unstable vaterite was transformed into calcite. In the presence of DMSO, the conversion rate of vaterite to calcite was the slowest, which was consistent with the largest measured interaction of DMSO molecules with the vaterite surface. However, a decrease in the rate of vaterite–calcite transformation was also observed for methanol and isopropanol when the CaCO_3_ particles were incubated in the reaction mixture for more than one hour. In addition, the strong interaction of isopropanol with the calcite’s surface may have been the reason for the formation of much smaller calcite particles after a one-day incubation period, in comparison to the CaCO_3_ crystals.

## 4. Conclusions

The influence of methanol, isopropanol and DMSO on the precipitation of calcium carbonate particles via the carbonation route, in the presence of ammonia as a CO_2_ absorption promoter, was investigated. In this work, the effect of the addition of organic solvents into the reaction mixture on the transformation of vaterite into calcite was studied for the first time. Furthermore, the precipitation of CaCO_3_ has not previously been carried out in DMSO solutions.

The presence of DMSO and methanol in the reaction mixture resulted in the slowing down of CaCO_3_ production, while the overall reaction rate was higher in the presence of isopropanol. Vaterite was the dominant form of precipitate, but the addition of organic solvents reduced the content of this polymorph immediately after the process was completed. What is more, the particle size was smaller when precipitation was carried out in the presence of the tested organic solvents. It was found that DMSO molecules interacted more strongly with the vaterite’s surface, while isopropanol showed a greater affinity with calcite. Based on the results obtained, it can be assumed that the effect of organic additions is twofold. During the formation of calcium carbonate particles, the dominant effect was associated with a decrease in the degree of dissociation of inorganic compounds in the solutions with organic solvents, which resulted in greater supersaturation and a decrease in the concentration of carbamic ions stabilizing vaterite. Therefore, the vaterite content of the precipitated calcium carbonate was reduced at the end of reaction. However, during the incubation period, the interaction of organic compounds with the surface of the vaterite and calcite played a major role, and the transformation of vaterite into calcite slowed down.

## Figures and Tables

**Table 1:** Selected physicochemical parameters of water, DMSO, methanol and isopropanol at 20 °C [32].

Solvent	Structure	Molar Mass (M) [g·mol^−1^]	Density (d) [g·cm^−3^]	Viscosity (η) [10^−3^ Pa·s]	Surface Tension (γ) [10^−3^ J·m^−2^]	Dielectric Constant (K) [−]	Dipole Moment (D) [−]
Water	H_2_O	18.02	0.998	0.891	71.98	78.5	1.85
DMSO	(CH_3_)_2_S=O	78.13	1.092	1.996	43.00	46.7	3.90
MeOH	CH_3_OH	32.04	0.791	0.545	22.07	32.6	1.60
iPrOH	(CH_3_)_2_CHOH	60.1	0.785	2.073	18.30	19.9	1.66

**Table 2:** The initial pH (pH_i_), reaction times (t_r_) and the percentage sequestrations of calcium ions (*%*Ca*^2+^*) depending on the concentration of selected solvents.

Solvent	Concentration (*v*/*v*) [%]	pH_i_	t_r_ [min]	%Ca^2+^
Control	0	11.03	17.8	75%
DMSO	5	10.98	18.8	66%
	10	10.96	19.2	68%
	15	11.01	19.8	69%
	20	10.97	20.1	71%
MeOH	5	11.04	16.0	69%
	10	11.00	16.5	68%
	15	10.98	17.0	66%
	20	10.98	17.4	64%
iPrOH	5	10.96	14.0	69%
	10	10.93	13.5	70%
	15	10.94	13.0	71%
	20	10.95	12.5	73%

**Table 3:** The percentage vaterite content (X_V_) depending on the incubation time and the concentration of selected solvents.

Sample		Control	DMSO				MeOH				iPrOH			
		Concentration (*v*/*v*) [%]												
		0	5	10	15	20	5	10	15	20	5	10	15	20
Incubation Time [h]	0	94.6	92.0	90.0	90.1	86.5	94.6	93.6	90.7	90.5	94.4	92.0	88.4	81.5
	1	79.6	73.6	71.4	78.6	78.9	60.0	57.2	50.4	59.2	74.9	52.1	44.9	24.4
	4	5.2	6.2	6.5	7.1	8.3	6.1	5.7	5.2	6.5	5.5	5.6	5.8	5.9
	24	4.6	5.6	5.9	6.0	6.1	5.7	5.4	5.0	5.7	5.3	5.3	5.2	5.2

**Table 4:** The values of calculated capacity factors (*k’*) for the tested normal phase high-performance liquid chromatography (NP-HPLC) conditions.

Solvent	k’	
	Vaterite	Calcite
DMSO	2.58	2.21
MeOH	2.50	2.29
iPrOH	2.09	2.75

## Supplementary Materials

The following are available online at https://www.mdpi.com/1996-1944/13/12/2742/s1, Figure S1: Fingerprint regions of FTIR spectra and XRD patterns of CaCO_3_ precipitated from a mixture containing 5% (a,b), 10% (c,d) and 20% (e,f)) of methanol, Figure S2: Fingerprint regions of FTIR spectra and XRD patterns of CaCO_3_ precipitated from a mixture containing 5% (a,b), 10% (c,d), 15% (e,f) and 20% (g,h) of isopropanol, Figure S3: Fingerprint regions of FTIR spectra and XRD patterns of CaCO_3_ precipitated from a mixture containing 5% (a,b), 10% (c,d), 15% (e,f) and 20% (g,h) of DMSO, Figure S4: FTIR spectra of precipitated CaCO_3_ depending on solvent concentration and incubation time for DMSO (a), methanol (b) and isopropanol (c), Figure S5: Particles size distributions (PSDs) depending on incubation time and solvent concentration for process without additives (a) and with addition of methanol (b1)–(b4), isopropanol (c1)–(c4) and DMSO (d1)–(d4), Figure S6: The average particle size curves versus incubation time for DMSO (a), methanol (b) and isopropanol (c) depending on solvent concentration, Figure S7: SEM photographs of calcium carbonate particles obtained from an aqueous solution and filtered immediately after completion of the reaction (a) and after 1 (b), 4 (c) and 24 hour (d) incubation, Figure S8: SEM photographs of CaCO_3_ particles precipitated in the presence of 5% of DMSO (a), isopropanol (b) and methanol (c) and not incubated, Figure S9: SEM photographs of CaCO_3_ particles precipitated in the presence of 10% of DMSO (a), isopropanol (b) and methanol (c) and not incubated, Figure S10: SEM photographs of CaCO_3_ particles precipitated in the presence of 20% of DMSO (a), isopropanol (b) and methanol (c) and not incubated, Figure S11: NP-HPLC chromatograms for the investigated solvents in columns loaded with calcite (a) and vaterite (b) particles.
