# Supplementary material for: Precipitation and Transformation of Vaterite Calcium Carbonate in the Presence of Some Organic Solvents
Source: Materials (Basel). 2020 Jun 17;13(12):2742. doi: 10.3390/ma13122742 (PMC7344821; doi:10.3390/ma13122742)
Supplement: Supplementary file 1 [file materials-13-02742-s001.pdf]

materials

Supplementary Materials

Precipitation and Transformation of Vaterite Calcium Carbonate in the Presence of Some Organic Solvents

Donata Konopacka-Łyskawa, Natalia Czaplicka, Marcin Łapiński, Barbara Kościelska and Rafał Bray

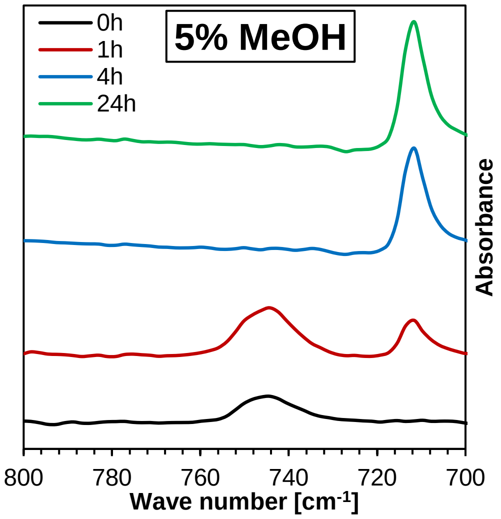

(**a**)

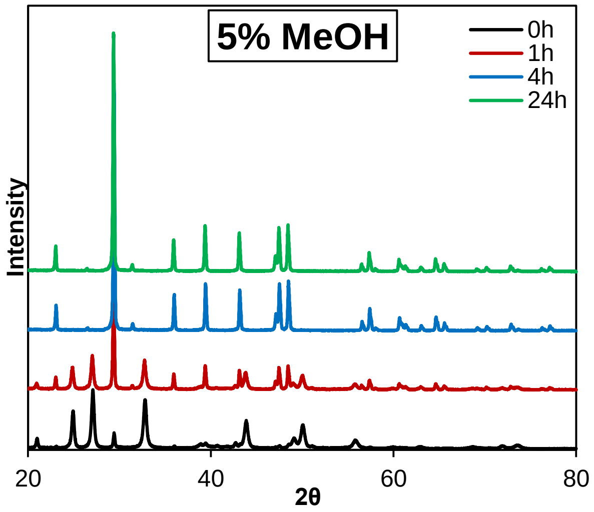

(**b**)

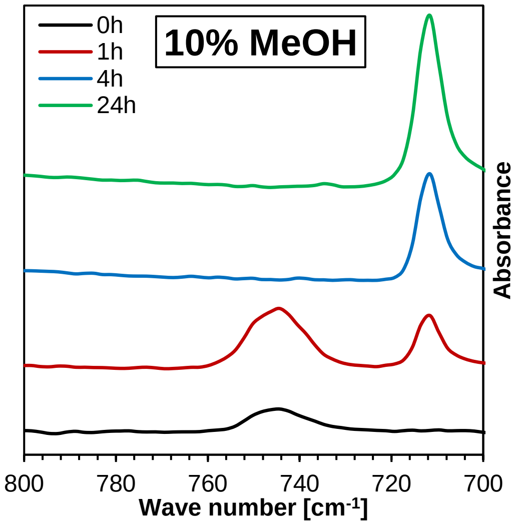

(**c**)

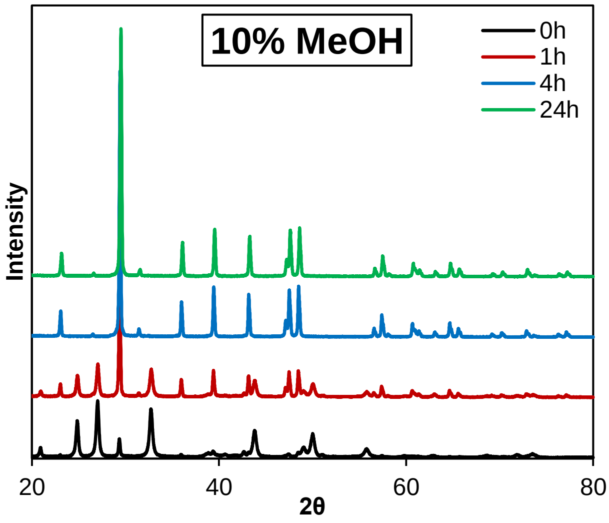

(**d**)

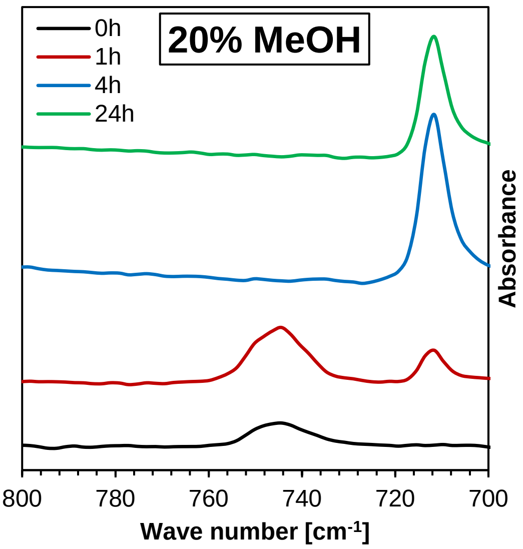

(**e**)

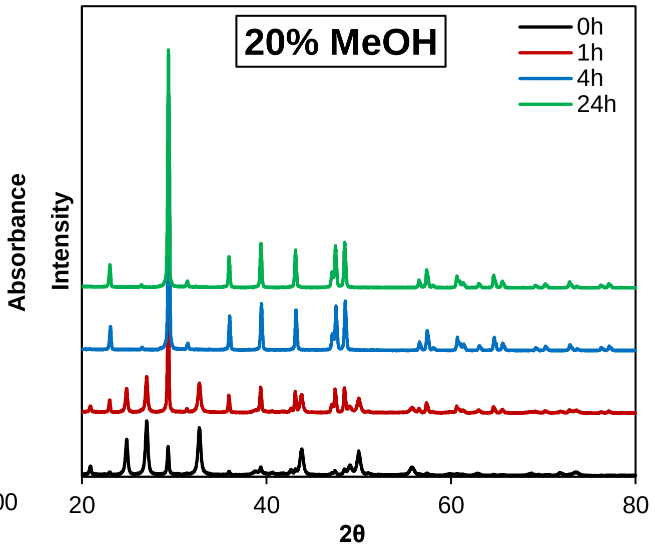

(**f**)

Figure S1. Fingerprint regions of FTIR spectra and XRD patterns of $CaCO_3$ precipitated from a mixture containing 5% (**a**,**b**), 10% (**c**,**d**) and 20% (**e**,**f**)) of methanol.

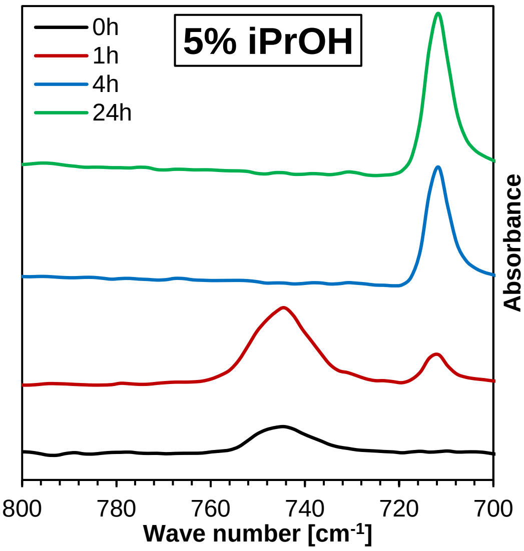

(**a**)

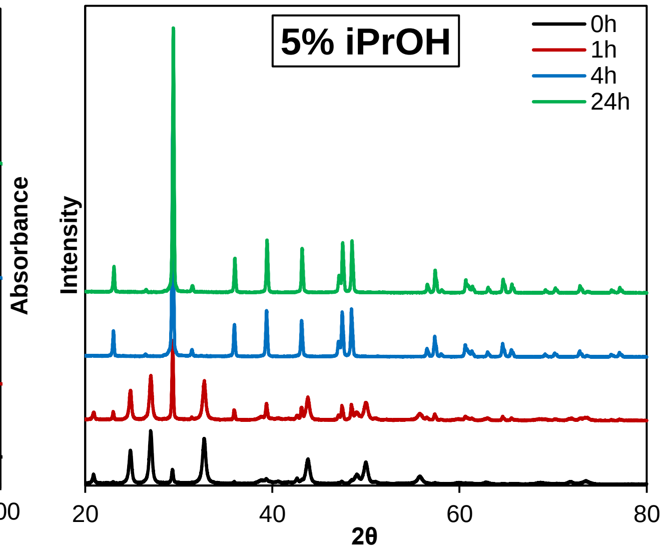

(**b**)

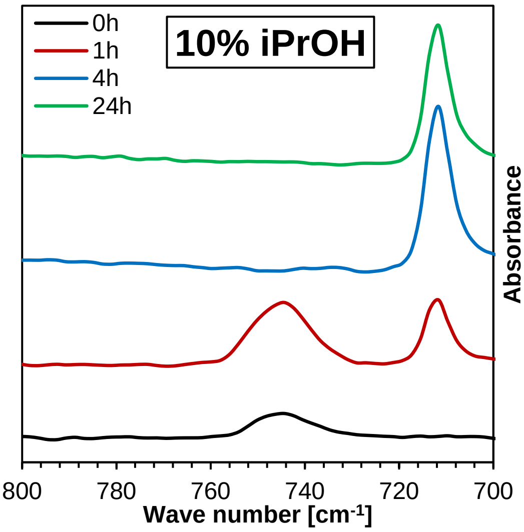

(**c**)

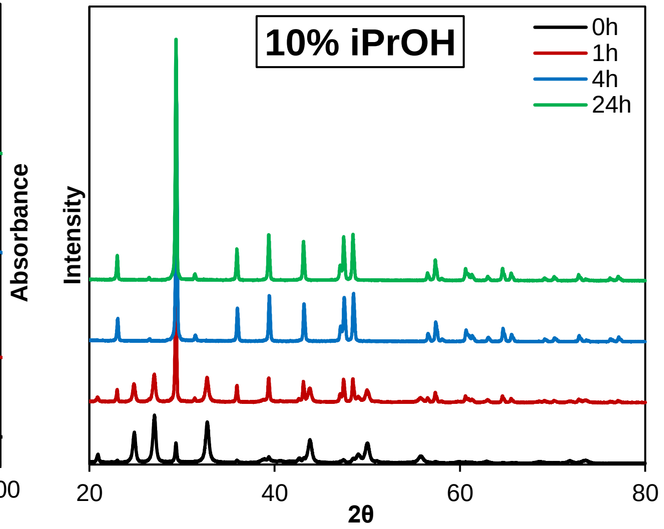

(**d**)

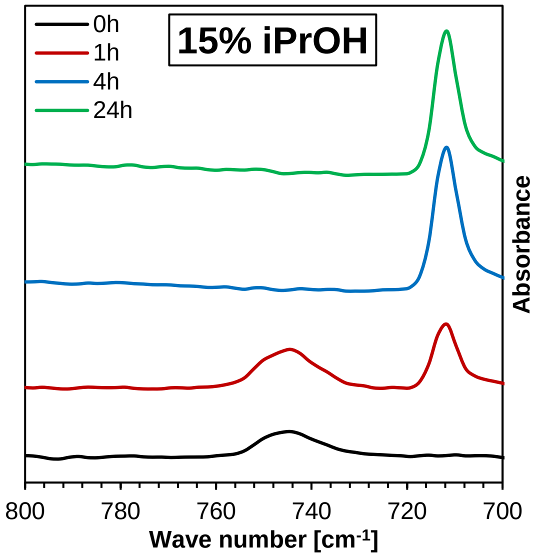

(**e**)

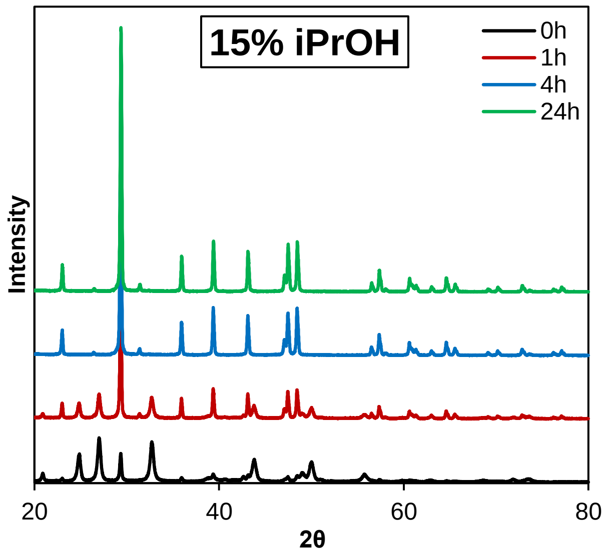

(**f**)

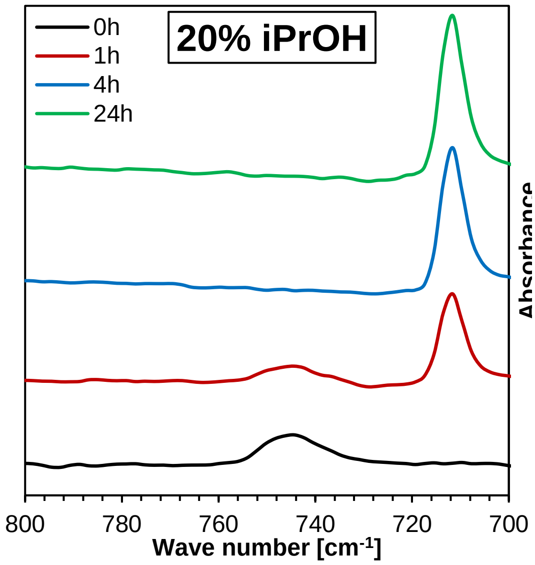

(**g**)

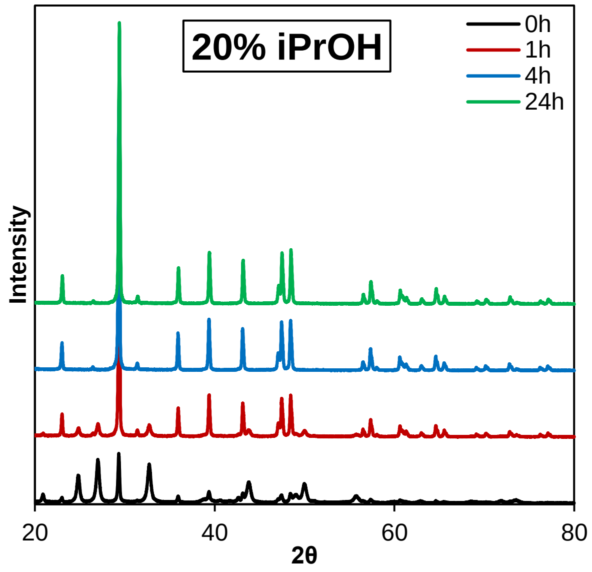

(**h**)

Figure S2. Fingerprint regions of FTIR spectra and XRD patterns of $CaCO_3$ precipitated from a mixture containing 5% (**a**,**b**), 10% (**c**,**d**), 15% (**e**,**f**) and 20% (**g**,**h**) of isopropanol.

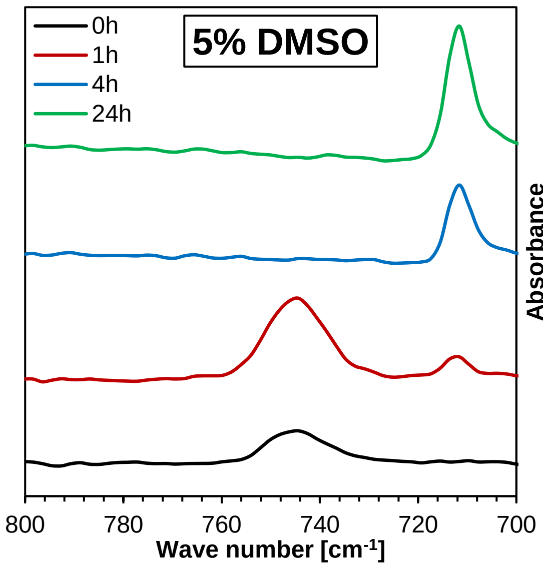
0h
1h
4h
24h
5% DMSO
800 780 760 740 720 700
Wave number [cm-1]
Absorbance

(**a**)

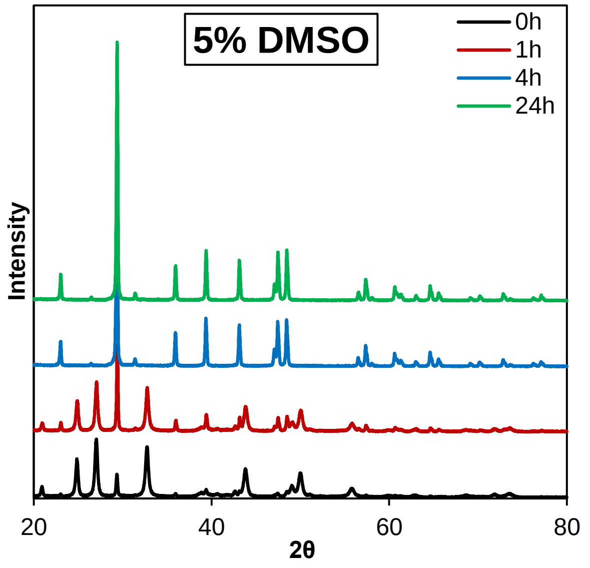
5% DMSO
0h
1h
4h
24h
Intensity
20 40 60 80
2θ

(**b**)

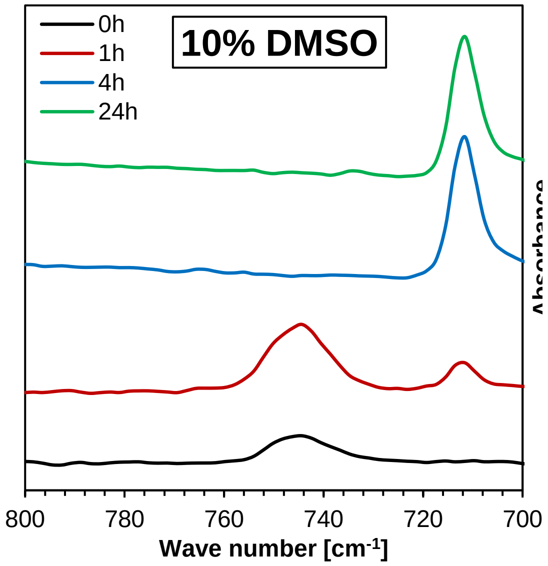
0h
1h
4h
24h
10% DMSO
800 780 760 740 720 700
Wave number [cm-1]

(**c**)

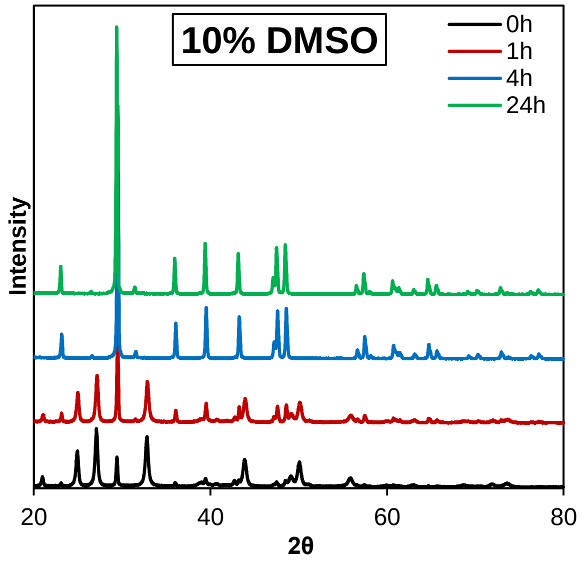
10% DMSO
0h
1h
4h
24h
Intensity
20 40 60 80
2θ

(**d**)

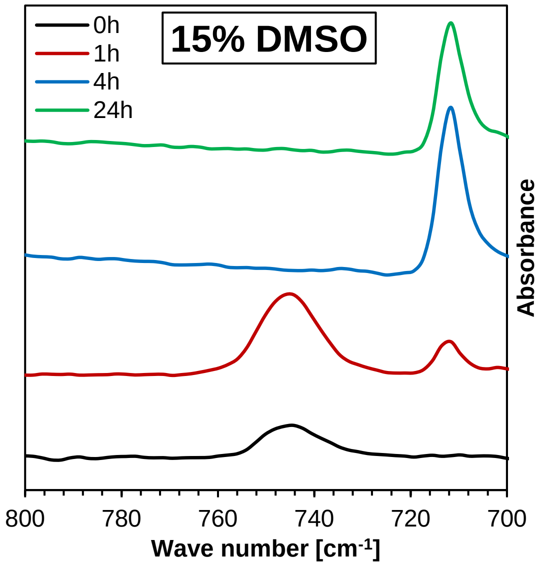
0h
1h
4h
24h
15% DMSO
800 780 760 740 720 700
Wave number [cm-1]
Absorbance

(**e**)

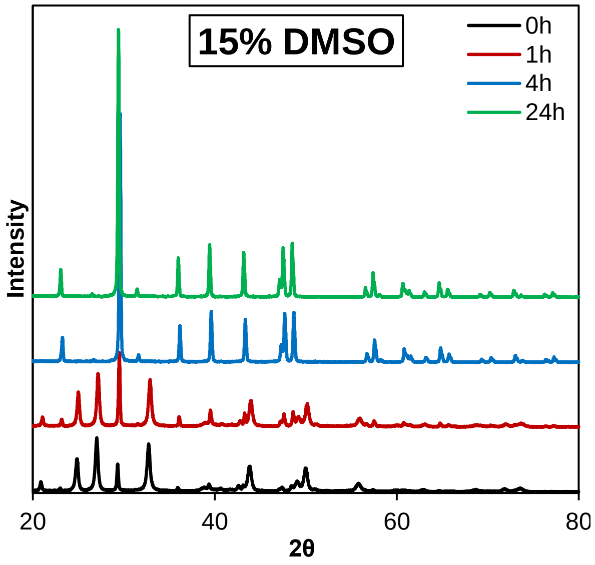
15% DMSO
0h
1h
4h
24h
Intensity
20 40 60 80
2θ

(**f**)

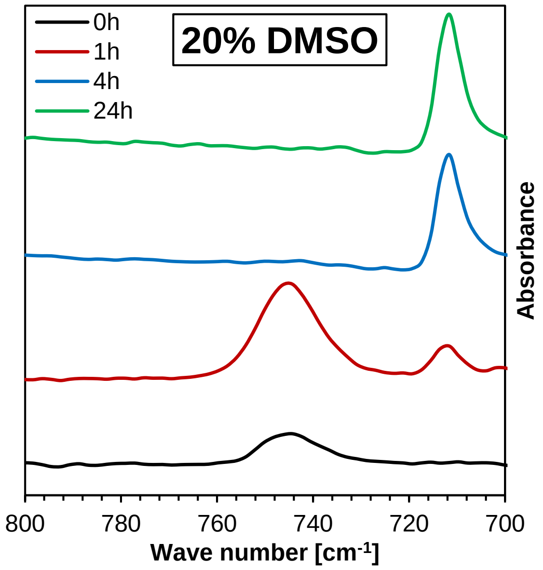

(**g**)

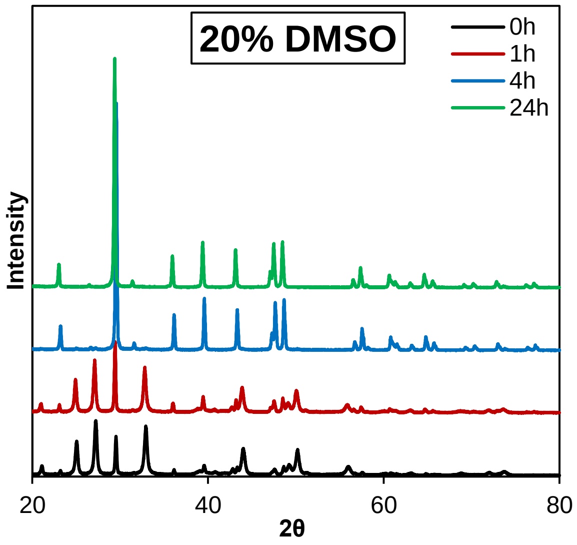

(**h**)

Figure S3. Fingerprint regions of FTIR spectra and XRD patterns of $CaCO_3$ precipitated from a mixture containing 5% (**a**,**b**), 10% (**c**,**d**), 15% (**e**,**f**) and 20% (**g**,**h**) of DMSO.

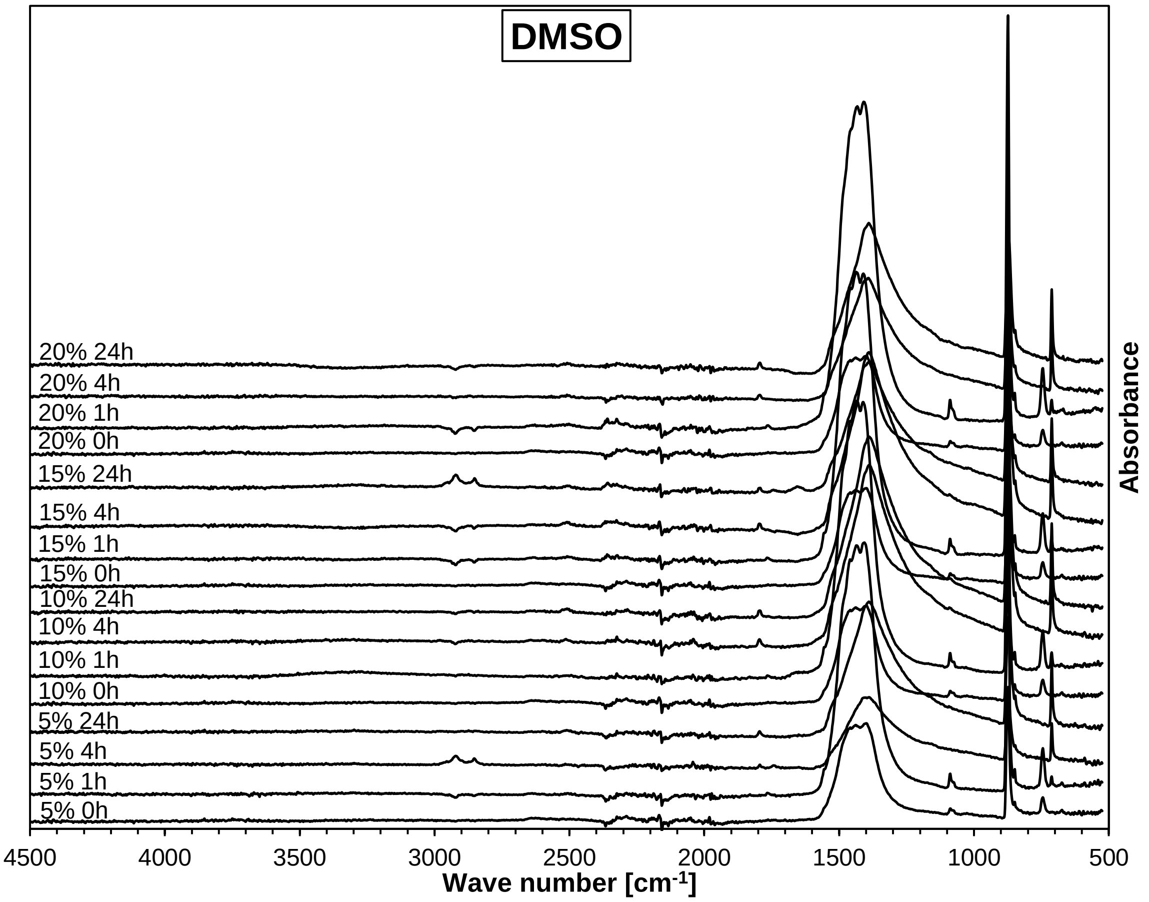

(**a**)

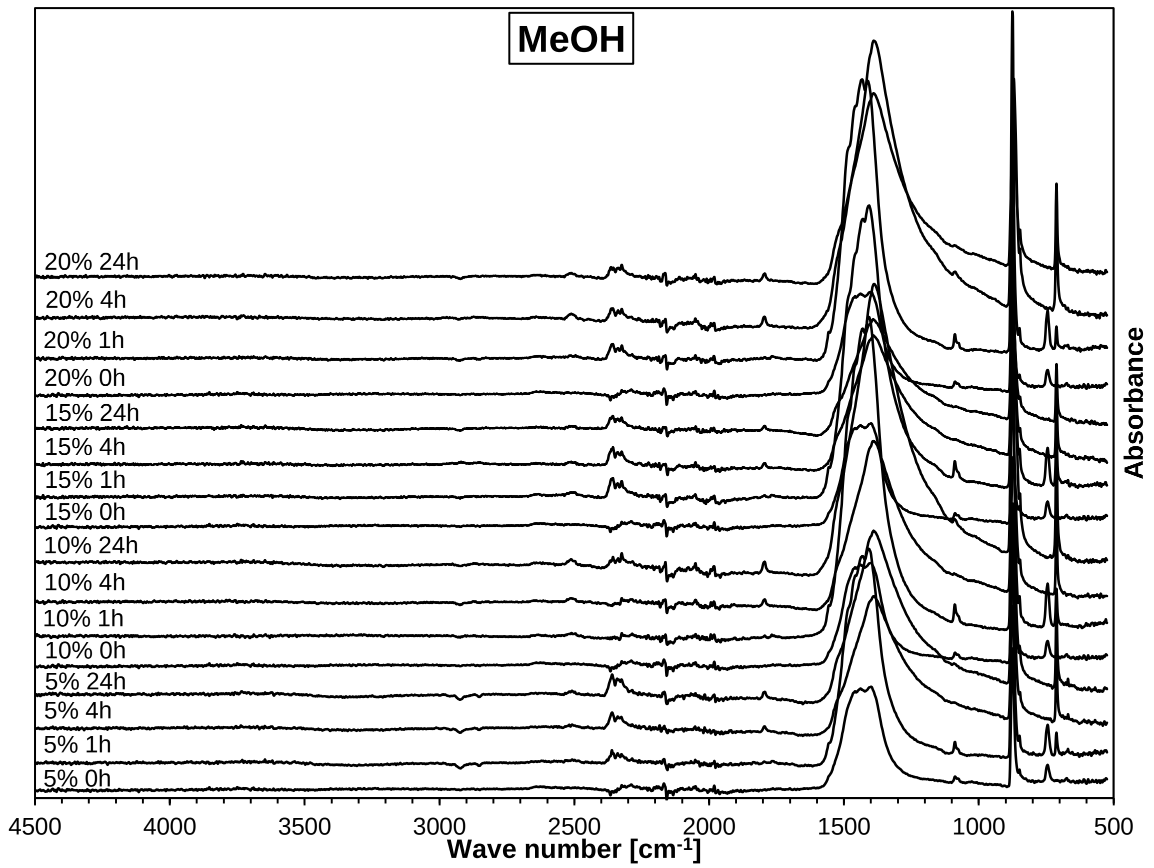

(**b**)

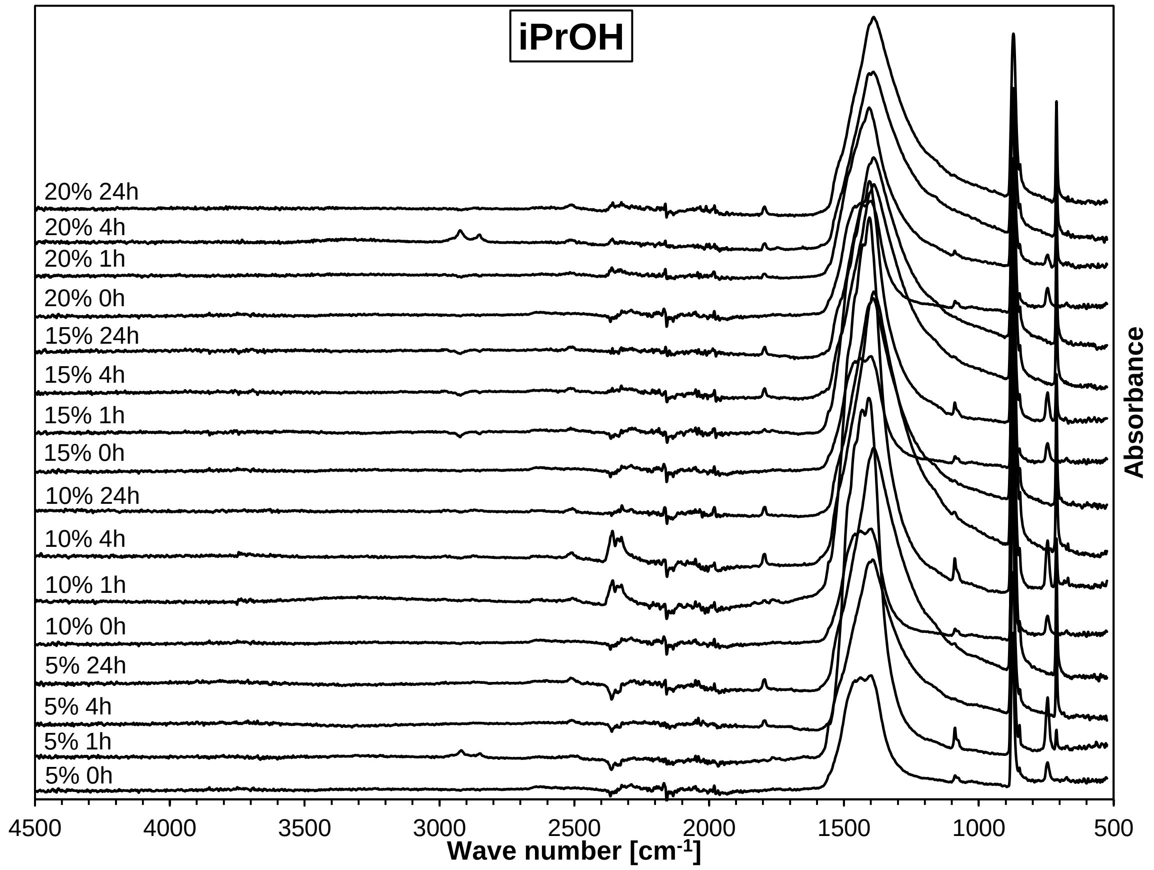

(**c**)

Figure S4. FTIR spectra of precipitated $CaCO_3$ depending on solvent concentration and incubation time for DMSO (**a**), methanol (**b**) and isopropanol (**c**).

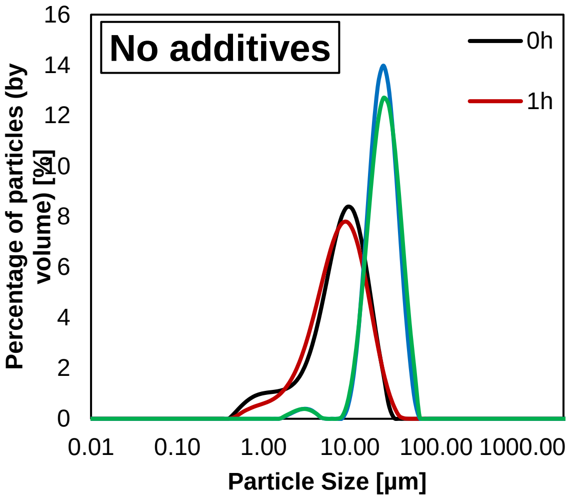
No additives
0h
1h
Percentage of particles (by volume) [%]
0
2
4
6
8
10
12
14
16
0.01
0.10
1.00
10.00
100.00
1000.00
Particle Size [μm]

(**a**)

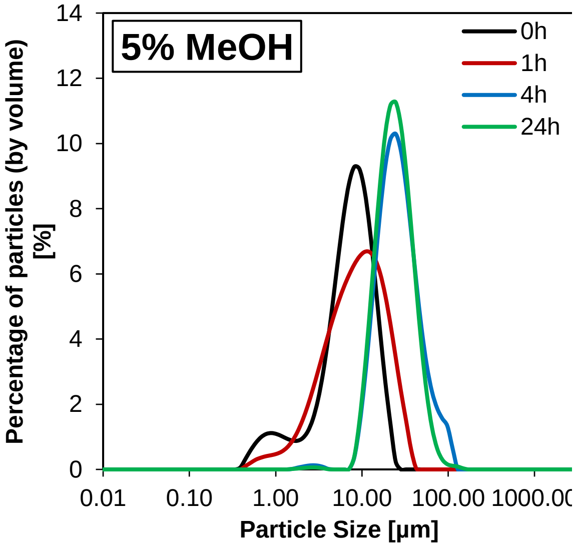
5% MeOH
0h
1h
4h
24h
Percentage of particles (by volume) [%]
0
2
4
6
8
10
12
14
0.01
0.10
1.00
10.00
100.00
Particle Size [μm]

(**b1**)

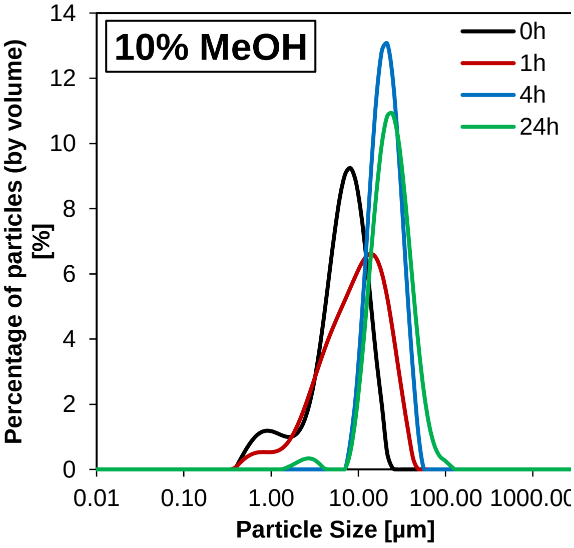
10% MeOH
0h
1h
4h
24h
Percentage of particles (by volume) [%]
0
2
4
6
8
10
12
14
0.01
0.10
1.00
10.00
100.00
Particle Size [μm]

(**b2**)

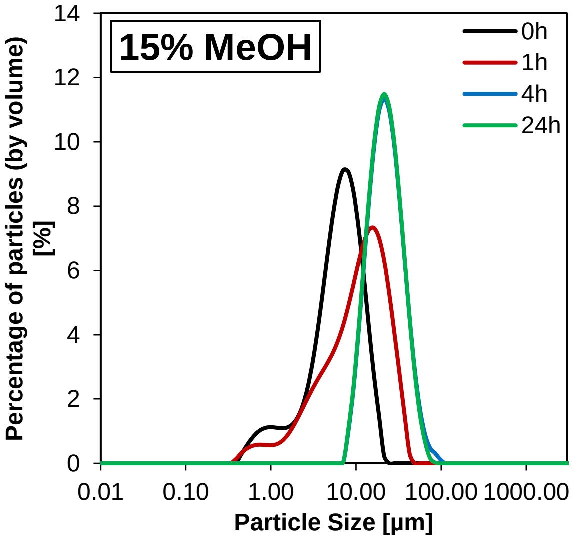
15% MeOH
0h
1h
4h
24h
Percentage of particles (by volume) [%]
0
2
4
6
8
10
12
14
0.01
0.10
1.00
10.00
100.00
1000.00
Particle Size [μm]

(**b3**)

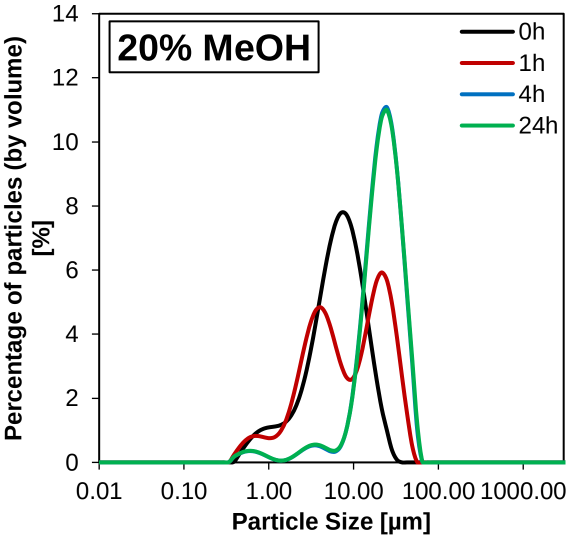
20% MeOH
0h
1h
4h
24h
Percentage of particles (by volume) [%]
0
2
4
6
8
10
12
14
0.01
0.10
1.00
10.00
100.00
1000.00
Particle Size [μm]

(**b4**)

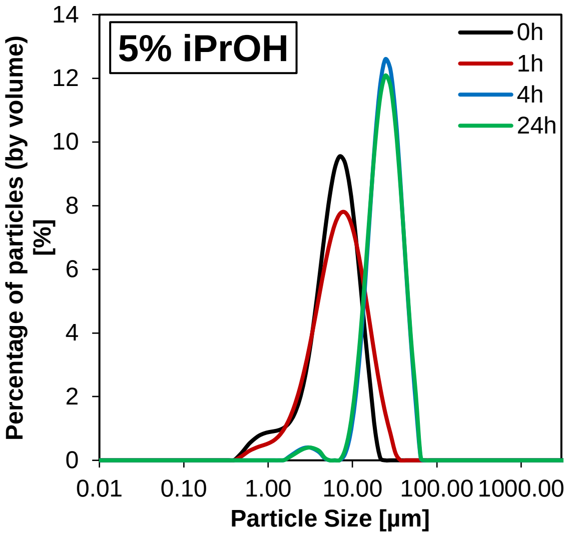
5% iPrOH
0h
1h
4h
24h
Percentage of particles (by volume) [%]
0
2
4
6
8
10
12
14
0.01
0.10
1.00
10.00
100.00
1000.00
Particle Size [µm]

(**c1**)

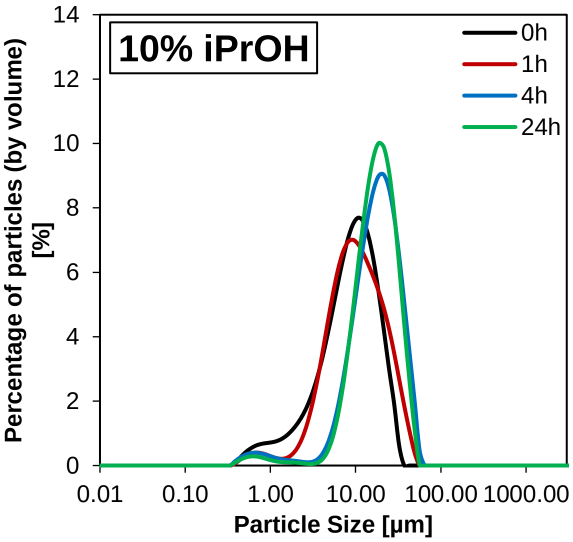
10% iPrOH
0h
1h
4h
24h
Percentage of particles (by volume) [%]
0
2
4
6
8
10
12
14
0.01
0.10
1.00
10.00
100.00
1000.00
Particle Size [µm]

(**c2**)

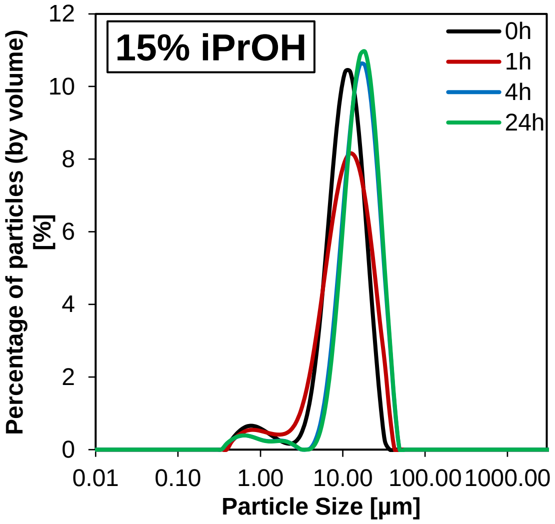
15% iPrOH
0h
1h
4h
24h
Percentage of particles (by volume) [%]
0
2
4
6
8
10
12
0.01
0.10
1.00
10.00
100.00
1000.00
Particle Size [µm]

(**c3**)

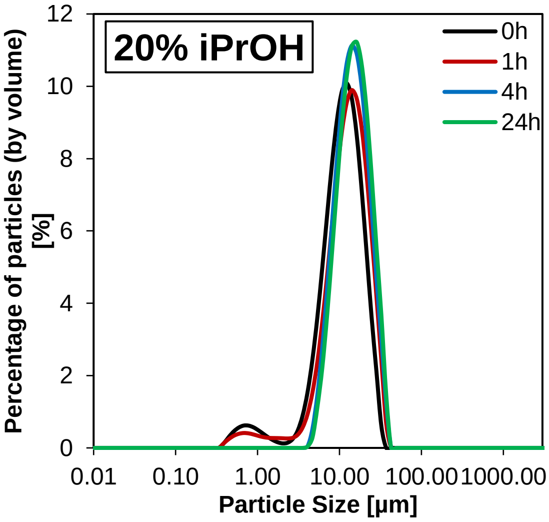
20% iPrOH
0h
1h
4h
24h
Percentage of particles (by volume) [%]
0
2
4
6
8
10
12
0.01
0.10
1.00
10.00
100.00
1000.00
Particle Size [µm]

(**c4**)

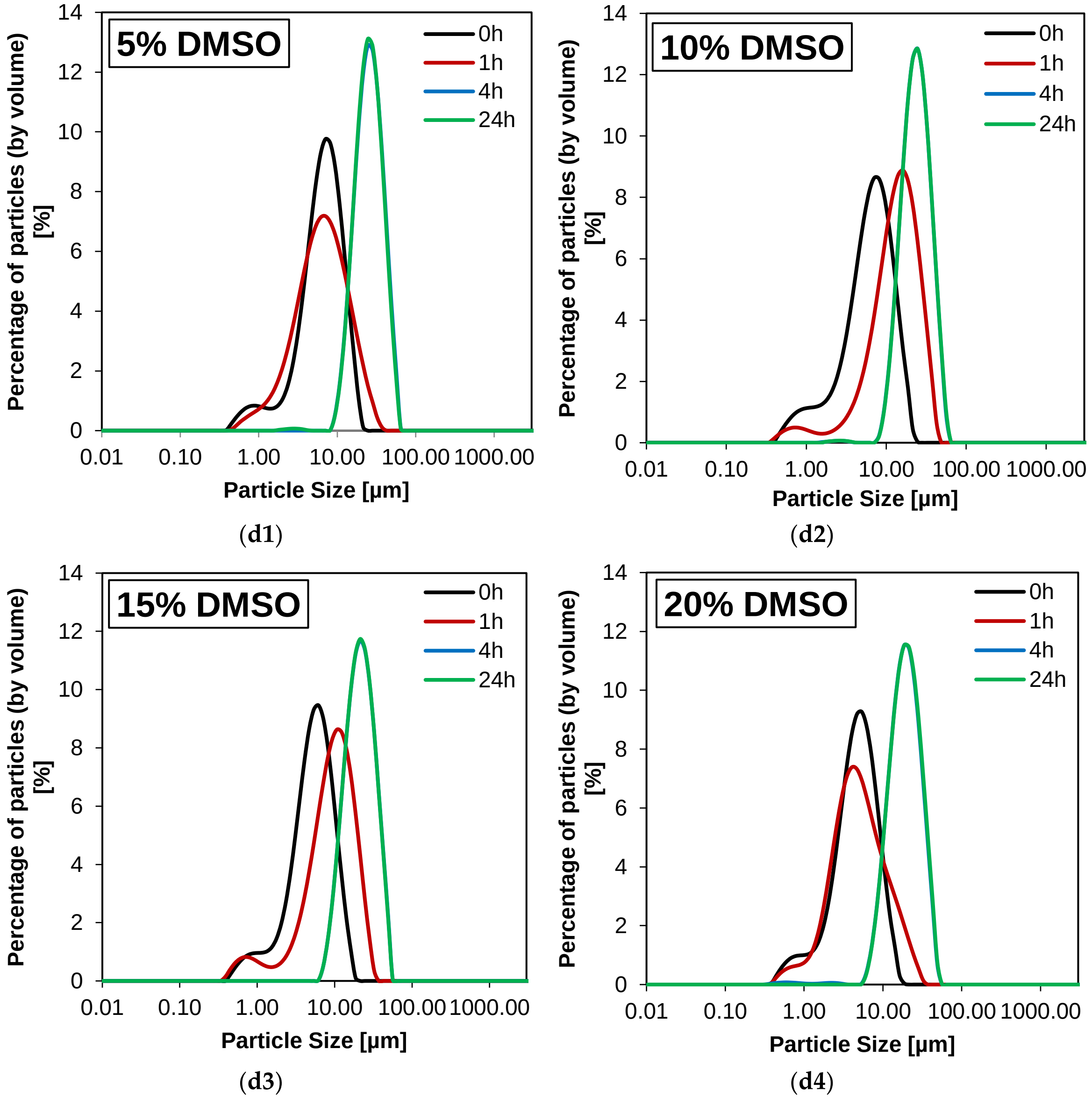

Figure S5. Particles size distributions (PSDs) depending on incubation time and solvent concentration for process without additives (**a**) and with addition of methanol (**b1**)–(**b4**), isopropanol (**c1**)–(**c4**) and DMSO (**d1**)–(**d4**).

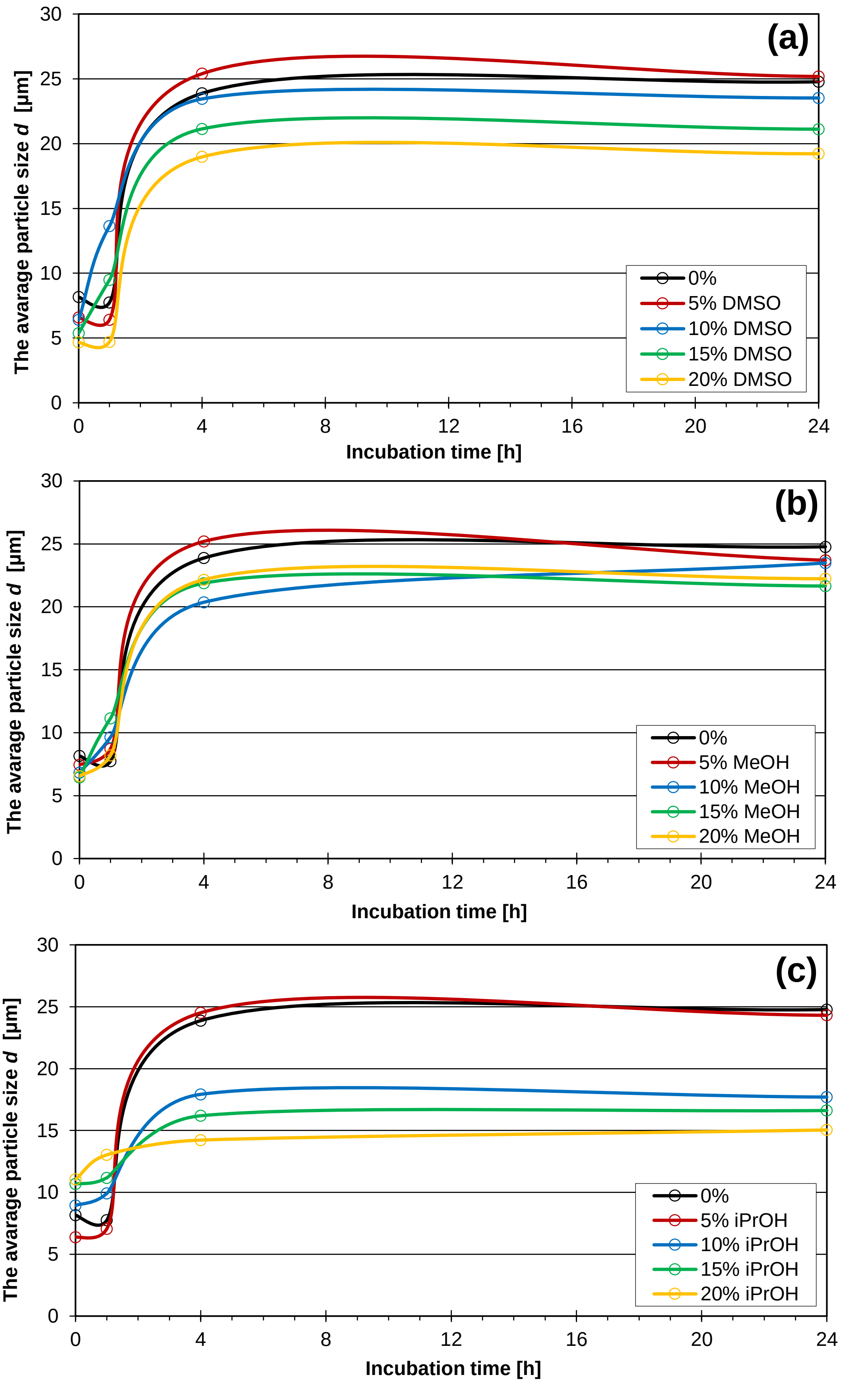

Figure S6. The average particle size curves versus incubation time for DMSO (**a**), methanol (**b**) and isopropanol (**c**) depending on solvent concentration.

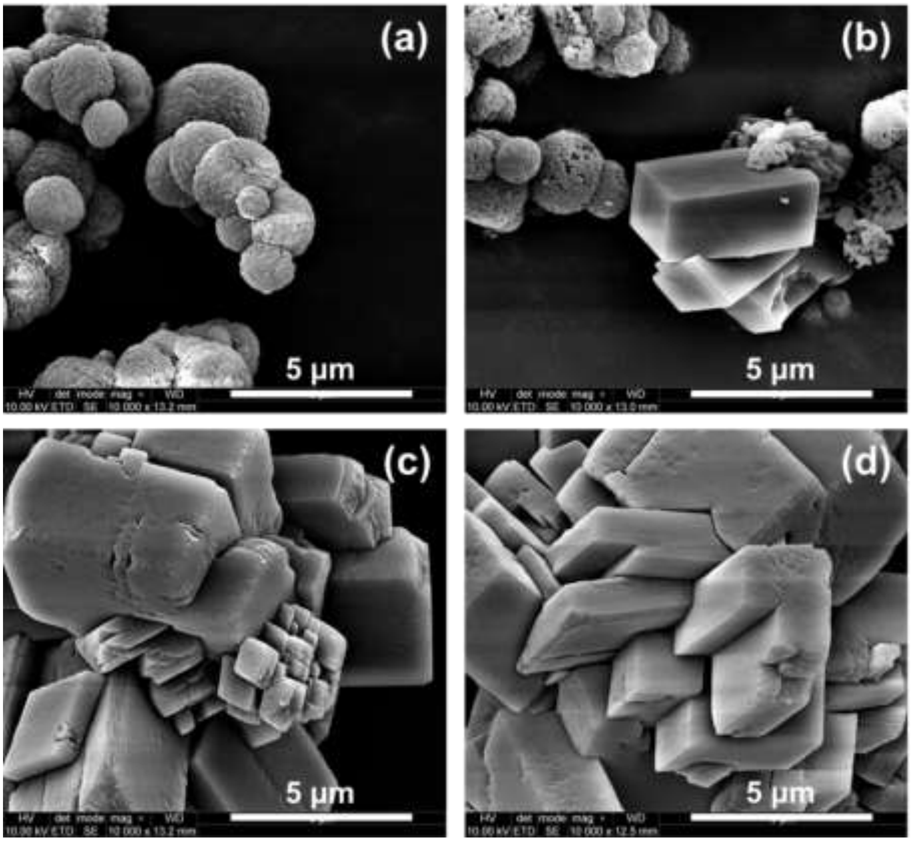

Figure S7. SEM photographs of calcium carbonate particles obtained from an aqueous solution and filtered immediately after completion of the reaction (**a**) and after 1 h (**b**), 4 h (**c**) and 24 h (**d**) incubation.

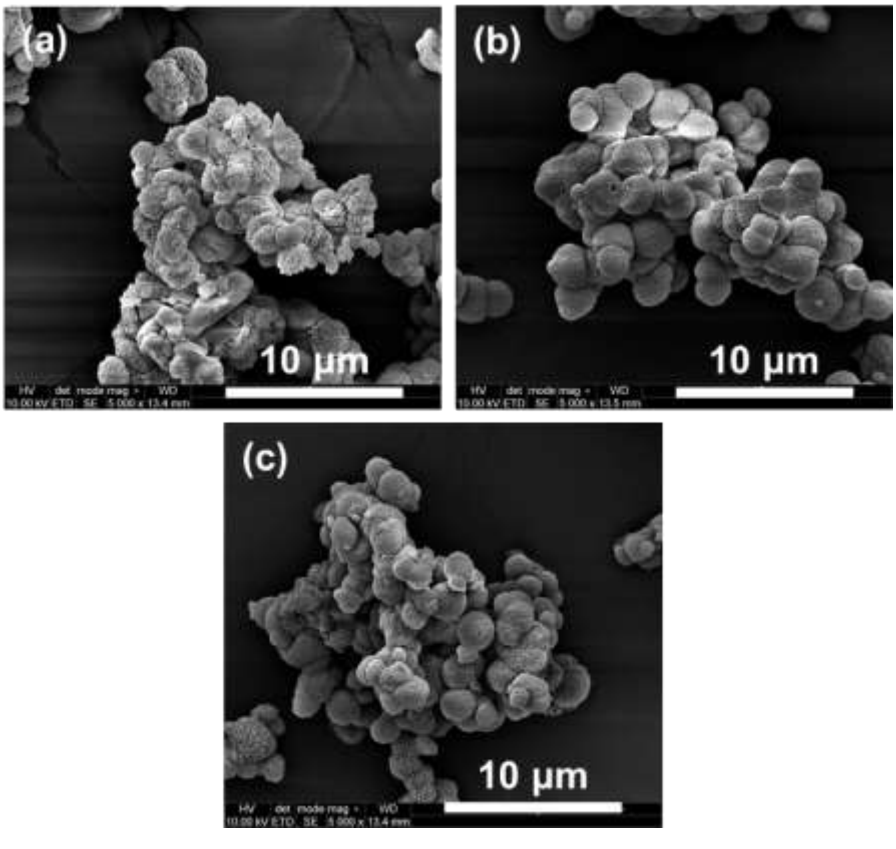

Figure S8. SEM photographs of $CaCO_3$ particles precipitated in the presence of 5% of DMSO (**a**), isopropanol (**b**) and methanol (**c**) and not incubated.

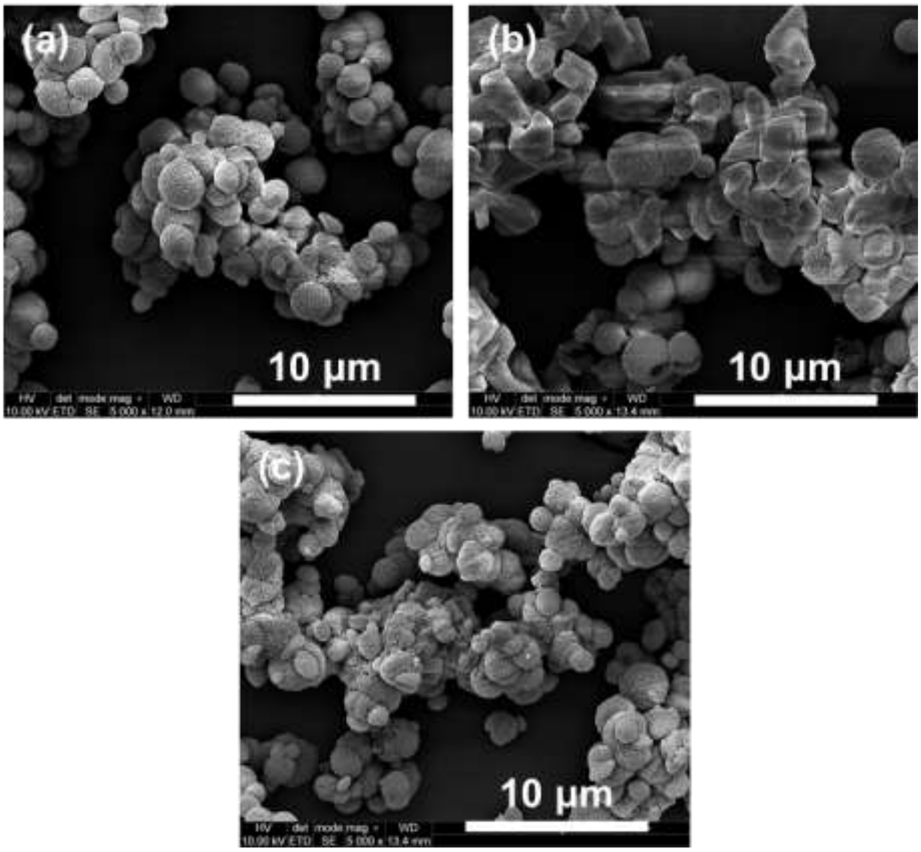

Figure S9. SEM photographs of $CaCO_3$ particles precipitated in the presence of 10% of DMSO (**a**), isopropanol (**b**) and methanol (**c**) and not incubated.

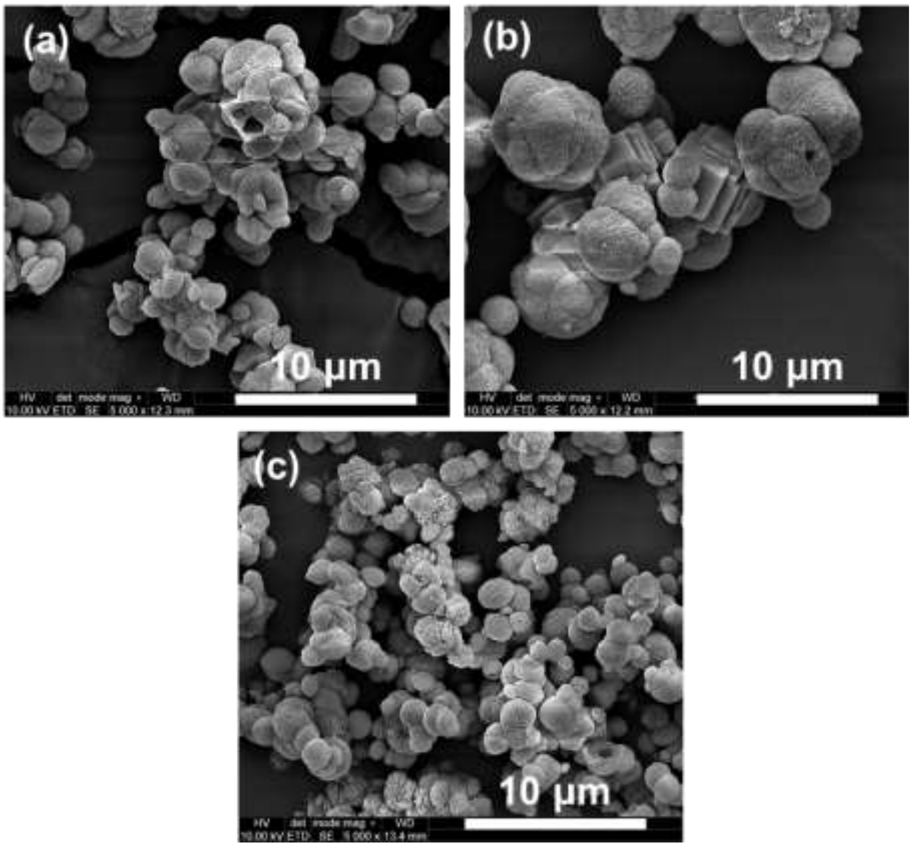

Figure S10. SEM photographs of $CaCO_3$ particles precipitated in the presence of 20% of DMSO (**a**), isopropanol (**b**) and methanol (**c**) and not incubated.

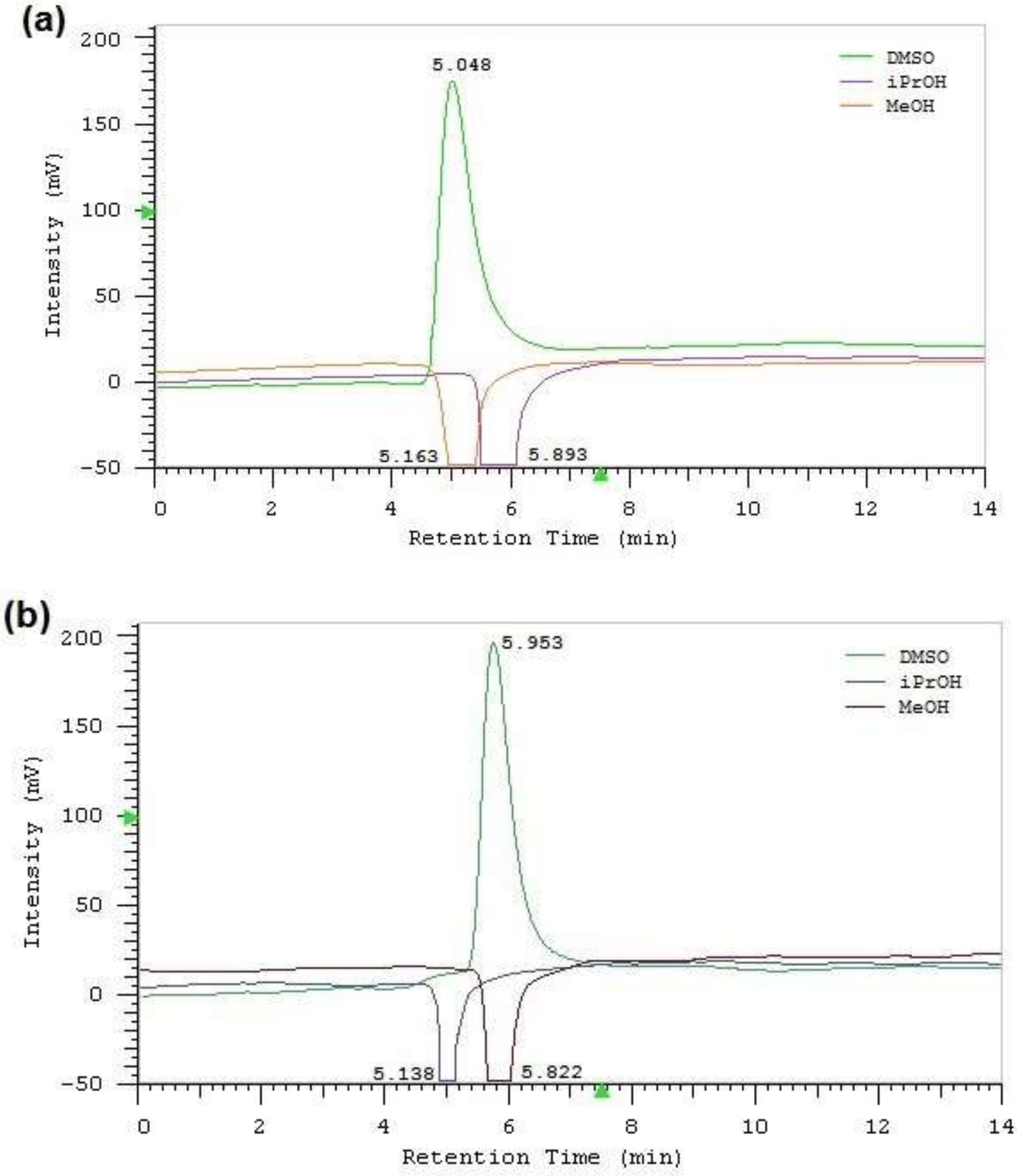

Figure S11. NP-HPLC chromatograms for the investigated solvents in columns loaded with calcite (**a**) and vaterite (**b**) particles.

© 2020 by the authors. Licensee MDPI, Basel, Switzerland. This article is an open access article distributed under the terms and conditions of the Creative Commons Attribution (CC BY) license (http://creativecommons.org/licenses/by/4.0/).
